# Cerebral Reorganization in Patients with Brachial Plexus Birth Injury and Residual Shoulder Problems

**DOI:** 10.3389/fneur.2016.00240

**Published:** 2016-12-23

**Authors:** Anders Björkman, Andreas Weibull, Hampus Svensson, Lars Dahlin

**Affiliations:** ^1^Department of Hand Surgery, Skåne University Hospital, Malmö, Sweden; ^2^Department of Radiation Physics, Skåne University Hospital, Malmö, Sweden; ^3^Department of Translational Medicine – Hand Surgery, Lund University, Malmö, Sweden

**Keywords:** brain, reorganization, brachial plexus, brachial plexus birth injury, nerve, children

## Abstract

The functional outcome after a brachial plexus birth injury (BPBI) is based on changes in the peripheral nerve and in the central nervous system. Most patients with a BPBI recover, but residual deficits in shoulder function are not uncommon. The aim of this study was to determine cerebral activation patterns in patients with BPBI and also residual symptoms from the shoulder. In seven patients (six females and one male, aged 17–23 years) with a BPBI and residual shoulder problems (Mallet score IV or lower), the cerebral response to active movement of the shoulder and elbow of the injured and healthy arm was monitored using functional magnetic resonance imaging at 3 T. Movements, i.e., shoulder rotation or elbow flexion and extension, of the injured side resulted in a more pronounced and more extended activation of the contralateral primary sensorimotor cortex compared to the activation seen after moving the healthy shoulder and elbow. In addition, moving the shoulder or elbow on the injured side resulted in increased activation in ipsilateral primary sensorimotor areas an also increased activation in associated sensorimotor areas, in both hemispheres, located further posterior in the parietal lobe, which are known to be important for integration of motor tasks and spatial aspects of motor control. Thus, in this preliminary study based on a small cohort, patients with BPBI and residual shoulder problems show reorganization in sensorimotor areas in both hemispheres of the brain. The increased activation in ipsilateral sensorimotor areas and in areas that deal with both integration of motor tasks and spatial aspects of motor control in both hemispheres indicates altered dynamics between the hemispheres, which may be a cerebral compensation for the injury.

## Introduction

Brachial plexus birth injury [BPBI; incidence 0.4–5.0 per 1,000 births (1, 2)] is caused by stretching or tearing of the brachial plexus, usually during vaginal delivery. High birth weight and shoulder dystocia are the most important risk factors (2, 3). Most children with BPBI recover spontaneously, but up to 30% may suffer from permanent disability (2, 4), and the number of patients with spontaneous recovery may have been overestimated (5). Following a BPBI, a cascade of processes in the peripheral nerve culminate in target muscle reinnervation (6). However, in cases with a severe injury, successful nerve regeneration may not be possible at all (7). In patients in whom regenerating axons reach the target muscles, there are still significant barriers to an optimal functional outcome, and the outcome is most often an incomplete reinnervation with a reduced number of functional motor units (8). In patients with residual symptoms after a BPBI, a pattern of recovery is often noted, but deficits are often seen in C5- to C7-innervated muscles. The degree of recovery of external rotation of the shoulder, elbow flexion, and supination at 3 months can be used as a predictor of which infants will retain functional deficits (9). Although external rotation of the shoulder and forearm supination are most affected and recover last, elbow flexion and shoulder abduction are the functional movements that often prove most challenging in patients with severe BPBI (9). Depending of the type of residual problems, surgical muscle transfer and osteotomy can improve function in patients with permanent disability (10). Even so, a number of patients have residual shoulder problems that restrict their daily life.

Evidence is accumulating to suggest that adaptations within the central nervous system are relevant for the clinical recovery following BPI in adults (8, 11–13). Some previous studies, using EMG, in patents with BPBI and residual symptoms has shown partial muscle reinnervation but poor clinical function—a condition termed developmental apraxia (14). This indicates a maladaptation of the cerebral motor network early in infancy following BPBI (15, 16). Thus, beyond peripheral nerve regeneration, plasticity in the central nervous system is involved in determining whether there is successful or failed functional recovery in patients with BPBI.

Our aim was to investigate cerebral activation patterns following activation of muscles controlling shoulder rotation and elbow flexion in young adults with BPBI and subjective shoulder problems.

## Materials and Methods

### Patients

All patients treated for BPBI at the Department of Hand Surgery, Skåne University Hospital, Malmö, Sweden between 2010 and 2011 were asked to participate. Inclusion criteria were as follows: (1) unilateral BPBI; (2) age between 15 and 25 years; (3) no previous reconstructive nerve surgery, i.e., neurolysis, nerve grafting, or neurotisation; (4) residual symptoms from the shoulder with a suboptimal Mallet score (17) (IV or lower) in one or more of the following variables: shoulder abduction, shoulder rotation, and hand to mouth; and (5) a minimum of 36 months since operation in patients operated with reconstruction to improve shoulder function. Exclusion criteria were as follows: (1) a history of neurological disease other than BPBI; (2) psychiatric disorders; and (3) contraindications to investigation by MRI.

Ten patients (eight females and two males) were identified, but one had moved abroad, and two did not want to undergo MRI investigation. Thus, seven patients (six females and one male) between 17 and 23 years of age were included in the study (Table 1). All patients had been meticulously followed since birth by specialists from the Departments of Paediatrics and Hand Surgery. At birth, they all had a normal hand function and a rapid initial recovery of some function in the biceps muscle, which indicates a nerve injury at trunk level. The probability of an avulsion injury of one or two of the nerve roots corresponding to the upper trunk seems to be low, which is also supported by the facts that none of the patients had a breech delivery.

**Table 1:** **Patient characteristics, Narakas group at birth, and Mallet score before and after reconstructive surgery in patients with brachial plexus birth injury**.

Patient no.	Gender	Age at functional magnetic resonance imaging (fMRI), years	Affected side	Narakas group	Mallet score before operation^a^	Age at surgery, years	Type of operation	Mallet score after operation^a^ (months after op.)
1	F	17	L	I	V/II/IV^b^	n/a	n/a	n/a
2	F	23	R	I	V/II/III	12	A, B, D	V/IV/V (130)
3	F	18	R	II	V/I/II	14	C, D	V/IV/V (46)
4	M	21	L	I	V/I/V	13	A, B, C	V/II/V (90)
5	F	23	L	I	IV/II/V	9	B, D	IV/IV/V (160)
6	F	17	L	I	IV/II/III	14	A, B, C, D	V/IV/V (36)
7	F	23	R	I	III/I/III	5 and 6	B, D	III/IV/IV (200)

*^a^Mallet score for shoulder abduction, external rotation of the shoulder, and hand to mouth*.

*A, resection of the coracoid process; B, lengthening of subscapular muscle; C, rotational osteotomy of humerus; D, transfer of latissimus dorsi muscle; n/a, not applicable*.

*^b^Patient not operated. Mallet score at the time of fMRI investigation*.

Six participants had been operated with reconstructive surgery in some form, although not nerve surgery, to improve shoulder function. None of the patients had been operated to improve elbow function. All the participants were classified according to the Narakas score at birth (18) and according to the Mallet score at the time of surgery and at follow-up (Table 1).

The study was approved by the ethical committee of Lund University (number 269/2008, 2009/728, additional approval 2011/23) and was conducted according to the tenets of the Declaration of Helsinki. All the participants gave written consent.

### Imaging

Functional magnetic resonance imaging (fMRI) was performed 36–200 months (mean 110 months) after surgery in six patients who were operated with reconstruction to improve shoulder function and in one additional patient with residual shoulder problems who had not been operated.

Functional magnetic resonance imaging was used to investigate cortical activation following active movement of the shoulder and elbow in a whole-body 3 T MR scanner (Tim-TRIO; Siemens Medical Solutions, Erlangen, Germany) equipped with a 12-channel head matrix coil. Initially, a high-resolution 3D anatomical scan was acquired with transversal slices oriented to form a plane through the anterior and posterior commissures.

Each patient was instructed to perform repetitive flexion/extension of the elbow joint or shoulder rotation (internal/external) at a constant pace during four 30-s periods interspersed with four 30-s periods of “rest.”

Sessions of motor activation were alternated with rest conditions of no stimuli in a block design. Each block was 30 s in length, and the experiment started with a rest condition. The same procedure was followed in the injured arm and uninjured arm in a pseudo-randomized order to avoid temporal bias, such as habituation effects and fatigue.

Blood oxygen level-dependent imaging was performed using a gradient echo–echo planar pulse sequence with an echo time of 30 ms, a repetition time of 2,000 ms, and a voxel resolution of 3 mm × 3 mm × 3 mm. After the scanning session, each patient was asked whether any complications had occurred, in order to ensure the use of proper data and to allow data to be excluded on reasonable grounds.

### Analysis

Evaluation of the fMRI data was performed using BrainVoyager QX 2.6 software (Brain Innovation B.V., Maastricht, the Netherlands). The functional data series was motion-corrected and spatially smoothed using a smoothing kernel width of 6 mm. The data series was then normalized to Talairach space (19) by co-registration to Talairach-processed anatomical data. Furthermore, low-frequency modulation below two cycles per session was suppressed. Activation maps were created by modeling of mixed effects using the general linear model. Resulting activation maps were visually inspected at a statistical threshold of *p* < 0.05, corrected for multiple comparisons by controlling the false discovery rate (20). Activation present at this threshold was subsequently evaluated at a statistical threshold of *p* < 0.01, uncorrected for multiple comparisons, to avoid cluster size bias when comparing different sessions. Statistical comparisons between groups were performed at *p* < 0.05, uncorrected. Activation clusters were located using Talairach coordinate standardization, and the corresponding Brodmann area (BA) (defined as the BA within 3 mm of the most significant voxel of each cluster) was defined using the Talairach client (21, 22) in applicable cases. This automated classification resulted in some clusters being located in BAs not generally considered to be part of the motor network.

When performing group analysis, the activation maps of patients with a left-side injury were flipped in the left-to-right direction to prevent substantial loss of power, a strategy that has already been used (23). Since the hemispheres in a single subject are neither functional nor anatomically equal, the spatial noise is likely to increase. The contribution of spatial noise is, however, minimized—as the data are smoothed both individually and in group analysis.

## Results

**Figure 1:**
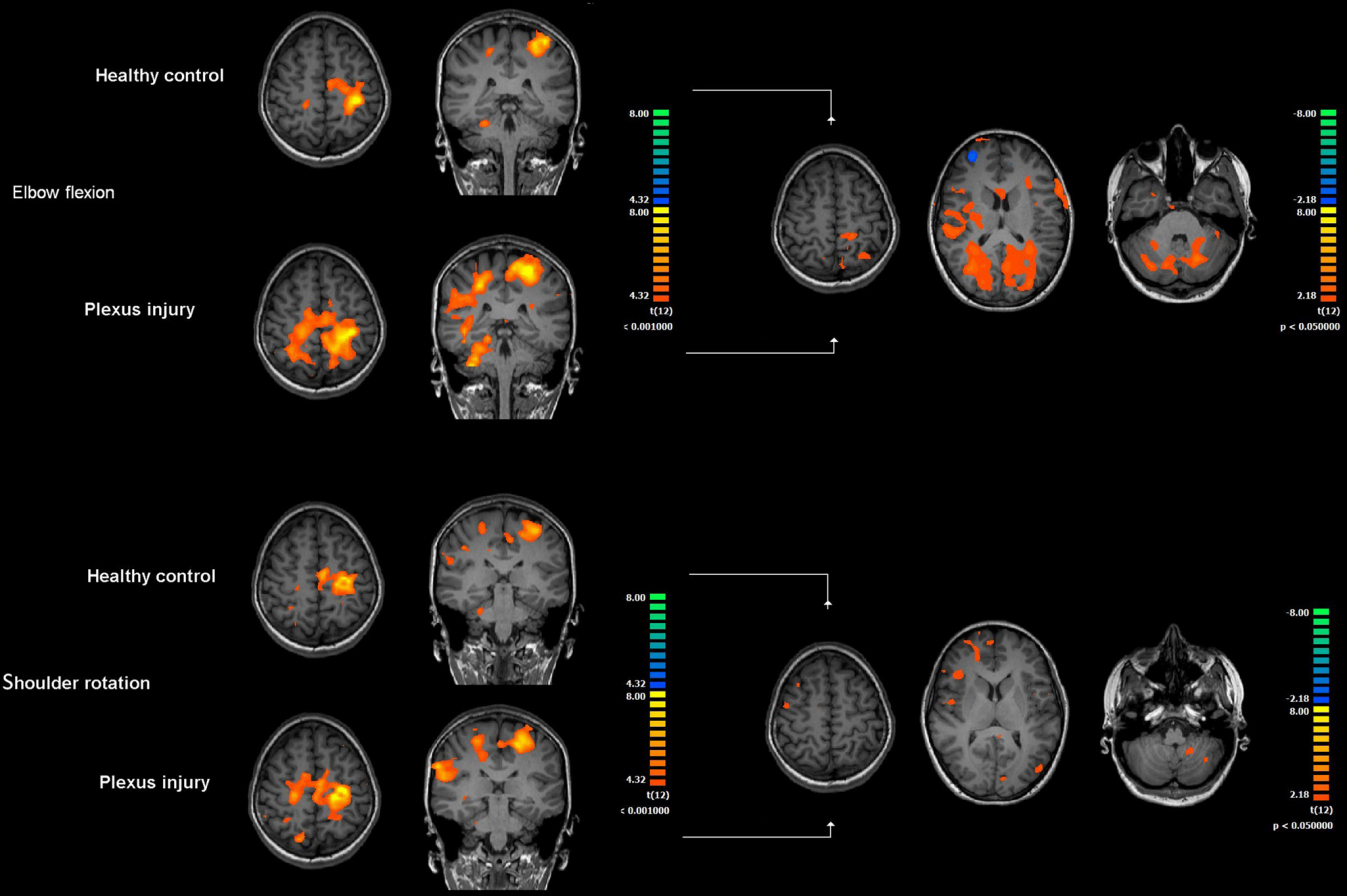
**Cerebral activation during motor stimulation in patients with brachial plexus birth injury**. The left-hand side of the figure shows brain activation during elbow flexion and extension and shoulder rotation compared to rest using the injured and healthy (control) arm, respectively. Activation patterns represent group results, and individual data have been shifted in the right–left direction so that the contralateral hemisphere in respect to motor stimulation is always the left hemisphere (conventionally to the right in radiological images). The right-hand side of the figure shows statistically significant differences (*p* < 0.05, uncorrected for multiple comparisons) in the activation pattern in the injured arm compared to the healthy arm. Elbow flexion resulted in significantly increased activation in the primary sensorimotor cortex in the contralateral hemisphere and in the ispilateral hemisphere compared to when moving the healthy arm (*p* < 0.05). Shoulder rotation on the injured side also resulted in increased activation in the primary sensorimotor cortex bilaterally, but this was not statistically significant.

The characteristics of the patients and the Narakas group at birth and Mallet score before and after any reconstructive surgery are presented in Table 1. All seven patients showed activation of the contralateral primary sensorimotor cortex during elbow movement and shoulder rotation of both the injured arm and the healthy arm. However, moving the injured arm, elbow flexion and extension, or shoulder rotation resulted in a more pronounced and more extended activation of the contralateral primary sensorimotor cortex and also the ipsilateral primary sensorimotor cortex than when moving the healthy arm (Figure 1). Furthermore, compared to when moving the healthy arm, moving the injured arm resulted in increased activation in associated sensorimotor areas, in both hemispheres, located further posterior in the parietal lobe. These areas are known to be important for integration of motor tasks and spatial aspects of motor control. In addition, increased activation was also seen in other regions, such as the supplementary motor area, secondary somatosensory cortex, the ipsilateral insula, and the cerebellum.

In the case of elbow movement, these differences were statistically significant (*p* < 0.05, uncorrected) when comparing the injured and healthy arms (Figure 1, right-hand side). During shoulder rotation, differences were less evident than changes seen during elbow movement, and they were not statistically significant at *p* < 0.05 (uncorrected) (Figure 1, right-hand side).

## Discussion

Patients suffering from unilateral BPBI and residual symptoms from the shoulder show increased activation in the primary sensorimotor cortex bilaterally and in associated sensorimotor areas, in both hemispheres, located further posterior in the parietal lobe which are known to be important for integration of motor tasks and spatial aspects of motor control, when using the injured arm compared to when using the healthy arm.

Normal use of the hands is highly dependent on interhemispheric control of motor and sensory areas in both brain hemispheres (24). In adults, a peripheral nerve injury in the forearm or a brachial plexus injury is known to result in substantial reorganization in sensorimotor areas in both brain hemispheres—and also in changes in functional connectivity between sensorimotor areas (11, 25). In addition, the return of sensory and motor functions after these injuries is often poor (26).

Interestingly, the cerebral response and clinical outcome after peripheral nerve injury have been shown to be highly dependent on age at injury (27, 28). The consequences of a neonatal peripheral nerve injury, such as BPBI, on the central nervous system in humans are largely unknown. However, studies on peripheral nerve injuries in neonatal rodents have shown that within days after injury most functional synapses turn into “silent synapses.” This is followed by a period of new synapse formation, i.e., reactive synaptogenesis, where new synapses are formed to support the damaged function (29). It is difficult to transform these findings to newborn humans with a BPBI, but a peripheral nerve injury, operated with nerve suture, in the forearm in children less than 13 years of age results in a cerebral activation in sensorimotor areas identical to that in healthy controls, in combination with an excellent return of sensory and motor function. On the other hand, a peripheral nerve injury in children older than 13 years results in a cerebral response similar to that seen in adults and a poor clinical outcome regarding return of sensory and motor functions (27). Given the fact that peripheral nerve injuries sustained at a young age have an excellent clinical outcome, patients with BPBI should have a good possibility of regaining function, and many patients with BPBI do have a complete functional recovery. Those who do not recover—as with the participants in the present study who had residual symptoms from their shoulder—probably have a more severe injury with extensive changes in afferent and efferent nerve signaling. This is supported by the present study, where fMRI showed cerebral changes that were more similar to what is seen in adults with peripheral nerve injuries, where activation in sensorimotor areas in both brain hemispheres is changed. Previous studies on adults with BPI have suggested altered interhemispheric dynamics, resulting in loss of the deactivation normally seen in ipsilateral sensorimotor areas (25). These results have been corroborated here in patients with BPBI and residual shoulder problems, who showed an increased activation in the primary sensorimotor cortex in the ipsilateral hemisphere. Furthermore, patients with BPBI also showed an increased activation in areas in the posterior parietal cortex in both hemispheres, which are known to be important for integration of motor tasks and spatial aspects of motor control. The mechanisms behind the change in neural activity in the ipsilateral primary sensorimotor cortex and the in associated sensorimotor areas found in this study are not clear. Previous studies in healthy humans have suggested a functional interhemispheric inhibition between the primary somatosensory cortices (30, 31), most likely mediated by transcallosal activation of inhibitory GABAergic interneurons (32). Thus, the changes in the ipsilateral hemisphere seen in our study could be driven by changes contralaterally, or they could be due to an altered balance between homologous parts of sensorimotor areas in both hemispheres. Furthermore, the increased activation seen in posterior parietal areas known to be important for spatial aspects of motor control and motor task integration also suggest that there may be a cerebral compensatory mechanism whereby more neurons are recruited to compensate for the impaired function in the arm.

All but one participant had Narakas I at birth, indicating shoulder and elbow problems. Over time, however, all the participants had improved spontaneously, and none of them had been operated to improve elbow function. At follow-up, none of them experienced any subjective problems from the elbow. On the other hand, all but one participant had been operated to improve shoulder function, and those who were operated also had an improved Mallet score although they still experienced problems from their shoulder to some extent. Interestingly, both shoulder rotation and elbow flexion of the injured arm resulted in cerebral changes in both brain hemispheres. However, these differences were only significant, compared to when using the healthy arm, after flexion and extension of the elbow. Keeping the small number of patients in mind, one possible explanation for this intriguing difference may be found in the dynamic capacity of the brain. Directly after the BPBI, there is a cerebral plasticity trying to compensate for the injury. The difference in clinical recovery in flexion and extension of the elbow and shoulder rotation in the patients may indicate a more severe injury to nerves supplying the shoulder. Six out of the seven patients underwent surgical reconstruction, at a later stage in life, to improve shoulder function, and even if they did not have nerve reconstructions, the surgical procedures performed are likely to result in a new period of cerebral plasticity where the neurons supplying the shoulder muscles adopt to the reconstructions done. This neural process can be detected as a clinical improvement in shoulder function. Thus, in the present study group, plasticity in neurons controlling elbow flexion and extension mainly take place at a very young age whereas the operative reconstructions to the shoulder are done at an older age where the dynamic capacity of the brain is diminished compared to what is the case in the period immediately after birth. This may result in a “smaller” reorganization, which is insufficient to improve shoulder rotation to a normal state.

Furthermore, the increased use of the hemisphere ipsilateral to the injury as well as areas in the posterior part of the parietal cortex bilaterally suggests that neurons in these areas have a more important role in the cerebral recovery processes in patients with BPBI than previously realized.

Our results were limited by the number of patients being examined. However, BPBI is rare, and there have been no previous studies focusing on cerebral changes in patients with BPBI and residual shoulder problems. Considering the long follow-up in this study, we believe that evaluation of seven patients can give valuable information, despite the small number. However, a study involving more patients who are randomized to different operative reconstructions is required to help answer two important questions: (1) is there a correlation between the cerebral changes, clinical deficits experienced by the patients, and the type of surgical reconstruction? and (2) does age at reconstruction affect clinical outcome and cerebral changes? Studies in neonatal rodents have shown that there is a critical time window for reactive, compensatory, synaptogenesis following a neonatal peripheral nerve injury (29). This time window is not known in humans; but since nerve repair before the age of 13 years results in perfect restitution of nerve function, reconstructive surgery for residual shoulder problems should, at least in theory, be done before the age of 13 years in order to optimize clinical outcome. These are important questions requiring further attention.

Many rehabilitation programs for patients with BPBI are purely empirical and are not based on current knowledge in neuroscience. The dynamic capacity of the nervous system creates possibilities, and recent studies have suggested the possibility of using specific interventions to accelerate axon regeneration and CNS plasticity, i.e., guided plasticity (33–35). It has been proposed that rehabilitation programs should be tailored individually according to the nerve injury and the functional problems experienced by the individual patient, to maximize the effects of guided plasticity (36). Further studies are needed to better understand the cerebral response to BPBI and to explore the potential therapeutic approach of guided plasticity in patients with BPBI.

## Author Contributions

AB and LD designed the study. AB, AW, HS, and LD assessed the patients and created a study database. AB, HS, and AW evaluated all fMRIs. AB, LD, and AW analyzed the results and wrote the manuscript. All the authors contributed to discussion of the results and read and approved the final version of the manuscript.

## Conflict of Interest Statement

The research was conducted in the absence of any commercial or financial relationship that could be construed as a potential conflict of interest.
